# The Healthy Brain Project: Embedding the Early Identification of Cognitive Impairment in Area Agencies on Aging

**DOI:** 10.1093/geroni/igaf122.2037

**Published:** 2025-12-31

**Authors:** Maria Brown, Sharon Brangman

**Affiliations:** Syracuse University, Syracuse, New York, United States; SUNY Upstate Medical University, Syracuse, New York, United States

## Abstract

Almost seven million older Americans live with Alzheimer’s disease and related dementias (ADRD). The Healthy Brain Project evaluates the practicality and impact of embedding evidence-based brief cognitive screens in case manager workflows in seven Area Agencies on Aging (AAAs) in Central New York State. The project team trained 50 case managers to administer an evidenced based brief cognitive screening tool (Mini-Cog™) and refer clients with scores (0-2 of 5) suggesting cognitive impairment for comprehensive assessment at the Central New York Center of Excellence in Alzheimer’s Disease (CEAD). Data collection began November 1, 2024, and concludes October 31, 2025. As of February 28, 2025 (presentation will include final data), AAAs have offered Mini-Cog™ screenings to 569 clients during routine client assessments; 301 (53%) have accepted screening. Of those screened, 117 (39%) had scores indicating potential cognitive impairment, 102 were offered referrals, 33 accepted a referral, 17 have been seen in the CEAD, and 14 have been diagnosed - more than half (8) were diagnosed with mild cognitive impairment. Early identification of cognitive impairment allows clinicians to identify treatable causes of dementia-like symptoms, provide access to treatments to help manage ADRD progression and symptoms, empower patients to make their own care decisions, and plan for supports to maximize independence and quality of life. Incorporating brief cognitive screens in routine AAA client assessments can identify older adults with previously unidentified and untreated cognitive issues who would benefit from these opportunities.

